# Systemic Candidiasis in Mice: New Insights From an Old Model

**DOI:** 10.3389/ffunb.2022.940884

**Published:** 2022-06-23

**Authors:** Berit Jungnickel, Ilse D. Jacobsen

**Affiliations:** ^1^ Department of Cell Biology, Institute of Biochemistry and Biophysics, Faculty of Biological Sciences, Friedrich Schiller University, Jena, Germany; ^2^ Research Group Microbial Immunology, Leibniz Institute for Natural Product Research and Infection Biology – Hans Knöll Institute, Jena, Germany; ^3^ Institute of Microbiology, Friedrich Schiller University, Jena, Germany; ^4^ Center for Sepsis Control and Care, Jena, Germany

**Keywords:** murine infection models, *Candida albicans*, filamentation, colonization, adaptive immunity

## Abstract

Animal models are essential to understand the pathophysiology of infections, to test novel antifungal compounds, and to determine the potential of adjunctive therapies, e.g. immune modulation. The murine model of systemic candidiasis induced by intravenous infection is technically straightforward, highly reproducible, and well-characterized. However, intravenous inoculation circumvents the necessity for the fungus to translocate across mucosal barriers, and the use of SPF mice that are immunologically naïve to *Candida* does not reflect the situation in human patients, in whom adaptive immune responses have been induced by mucosal colonization prior to infection. Therefore, mouse models that combine intestinal colonization and systemic infection have been developed, resulting in novel insights into host-fungal interactions and immunity. In this review, we summarize the main findings, current questions, and discuss how these might impact the translatability of results from mice to humans.

## Introduction

Systemic candidiasis is a term used to describe a spectrum of infections caused by *Candida* species disseminating *via* the blood stream to a range of internal organs, including liver, brain, kidneys, heart and eyes. It includes candidemia, the presence of fungi in the blood, deep-seated candidiasis affecting one or few internal organs (e.g. hepatosplenic candidiasis), or disseminated infection affecting several organs in different parts of the body (McCarty et al., 2021). Systemic candidiasis is a life-threatening condition that despite antifungal treatment still results in relatively high mortality rates of 20-40% (Cornely et al., 2020). Although a significant proportion of infections are caused by non-albicans species such as *C. glabrata*, *C. tropicalis*, *C. parapsilosis*, and *C. krusei*, *C. albicans* is the most common cause of systemic candidiasis worldwide (Guinea, 2014). In contrast to *C. tropicalis*, *C. parapsilosis*, and *C. krusei*, which can be isolated from environmental samples, *C. albicans* is nearly exclusively found in association with warm-blooded animals (Odds, 1988). In humans, it is transmitted vertically from mother to child and colonizes the oral, urogenital, and intestinal mucosa (Ghannoum et al., 2010; Drell et al., 2013; Nash et al., 2017). *C. albicans* is one of the most abundant members of the intestinal mycobiome, and its prevalence in the Human Microbiome Project healthy cohort was >60% (Nash et al., 2017). Most cases of systemic candidiasis by *C. albicans* are caused by strains that colonized the mucosal surfaces of the individuals before disease onset, with the gut being one of the main reservoirs for the fungus (Miranda et al., 2009; Zhai et al., 2020).

Animal models are essential to understand the pathophysiology of infections, to test novel antifungal compounds, and to determine the potential of adjunctive therapies, e.g. immune modulation. Candidiasis occurs in a range of domestic and wild animals (Seyedmousavi et al., 2018), and can be easily induced in laboratory animals by injecting yeast cells into the blood stream. Due to practical considerations, such as ease of husbandry and handling, availability of genetically defined and genetically modified strains, and reagents for molecular analyses, mice are predominantly used as model host for systemic candidiasis (Szabo and MacCallum, 2011). The murine model of systemic candidiasis induced by intravenous infection is technically straightforward, highly reproducible, and well-characterized (Szabo and MacCallum, 2011; MacCallum, 2013). However, intravenous inoculation circumvents the necessity for the fungus to translocate across mucosal barriers (Koh et al., 2008). Furthermore, the host is challenged by a single bolus of pathogens, which might not reflect continuous translocation over a prolonged period of time. To address this, a murine model of gastrointestinal colonization with induced translocation has been developed by A. Y. Koh in the lab of G. B. Pier. The model requires antibiotic treatment to achieve colonization levels that are sufficient to sustain translocation rates leading to clinical disease (Koh et al., 2008). In addition, both intestinal barrier dysfunction and neutropenia are necessary to induce translocation, consistent with older reports (Umenai et al., 1979; Mellado et al., 2000). Finally, the number of fungi translocating as effective infectious dose cannot be defined (Koh et al., 2008; Koh, 2013). Thereby, this model is substantially more complex than the intravenous infection model, more difficult to standardize, and it does not allow to investigate candidiasis in immunocompetent hosts. While immunosuppression and gut barrier damage increase the risk of systemic candidiasis in humans, they are not essential. Thus, this model represents only one group of patients at risk of systemic candidiasis. Nonetheless, the colonization-dissemination model was essential for the identification of host defense systems preventing translocation and subsequent candidiasis. Furthermore, it elucidated the route of spread from the gut: While the kidneys are the main target organ in the murine intravenous infection model, induced dissemination from the gut results in fungi predominantly entering the liver *via* the hepatic portal vein (MacCallum and Odds, 2005; Koh et al., 2008; Koh, 2013).

Although the two-step colonization-dissemination model as developed by A. Y. Koh is not used routinely to study systemic candidiasis, it provided important basic information that, together with the efforts of others, e.g. the Huffnagle lab (Mason et al., 2012), led to the establishment of *C. albicans* intestinal colonization models. These models were used to investigate how the host immune system responds to *Candida* colonization, and recently were combined with intravenous infection to investigate if and how colonization affects subsequent systemic candidiasis.

## *Candida* Colonization Affects Systemic Candidiasis

### Why Is Fungal Colonization Important?

The profound impact of microbial colonization on the mammalian immune system has been well established since germ free mice were used on a larger scale in the 1960s [reviewed in (Smith et al., 2007)]. In the last decades multiple mechanisms by which bacteria shape the development of the immune system and homeostasis were identified (Hooper et al., 2012). In comparison, very little was known about the role of fungi as part of the microbiota, for which the term mycobiota was coined. One reason is the lower abundance of fungi compared to bacteria in the gut (Huffnagle and Noverr, 2013; Nash et al., 2017), and the necessity to develop specific methods to analyze mycobiota composition (reviewed in (Li et al., 2019). However, specific pattern recognition receptors (especially C-type lectins) evolved to recognize fungi (Ward and Vyas, 2020), suggesting that fungi could have specific effects on host immunity. The first evidence supporting this notion came from observations that the titers of antibodies reactive to *S. cerevisiae* mannan (ASCA) were elevated in patients with Crohn’s disease or other immune mediated diseases (Schaffer et al., 2007). More recently, C-type lectins were found to be important for controlling fungal-driven inflammation in colitis models (Iliev et al., 2012; Wang et al., 2016). Lack of CX3CR1+ mononuclear phagocytes affects gut mycobiome composition and results in colitis in mice, and CX3CR1 defects are found in some Crohn’s disease patients (Leonardi et al., 2018). In mice, mycobiota changes induced by antifungal treatment were found to exacerbate both colitis and allergic airway disease, which involves gut-resident CX3CR1+ mononuclear phagocytes (Wheeler et al., 2016; Li et al., 2018; Skalski et al., 2018). In the absence of bacteria, however, commensal fungi provide an essential stimulus for immune maturation (Jiang et al., 2017).

### Immunological Responses to *C. albicans* Colonization

Most of the studies mentioned above used or included *Candida*, often *C. albicans*, as model fungi for intestinal colonization. *C. albicans* colonization furthermore induces robust Th17 responses in mice (Leonardi et al., 2018; Shao et al., 2019) and is the main inducer of Th17 cells in humans (Bacher et al., 2019). Importantly, these reactions are not limited to mucosal sites as reactive Th17 cells increase in the spleen (Shao et al., 2019). Th17 immunity has a crucial role in maintaining oral epithelial barriers and preventing oral candidiasis (Gaffen and Moutsopoulos, 2020) and IL-17 is important for defense against systemic candidiasis (Huang et al., 2004; Bär et al., 2014; Ramani et al., 2016). In addition, colonization induces antibody responses in mice and humans (Pitarch et al., 2006; Luo et al., 2016; Huertas et al., 2017; Doron et al., 2021), and antibodies occurring in human serum are able to increase interaction of phagocytes with *C. albicans*, and to reduce adhesion to and damage of epithelial cells by the fungus (Wich et al., 2021). In summary, mucosal colonization with *C. albicans* induces both local and systemic responses. Since most cases of systemic candidiasis by *C. albicans* are caused by endogenous strains that colonized the mucosal surfaces (Zhai et al., 2020), for humans it can be assumed that systemic infection with *C. albicans* occurs after some level of adaptive immunity has already been established. This is in stark contrast to the intravenous infection model using specific-pathogen free (SPF) mice. Laboratory mice bred and kept under strict hygiene regimens are usually not colonized with *Candida albicans* (Iliev et al., 2012; Wheeler et al., 2016; Skalski et al., 2018; Doron et al., 2019). Thus, SPF mice can be considered immunologically naïve to *Candida*.

### Impact of Colonization on Systemic Candidiasis in Mice

Since both Th17 responses and the systemic antibodies induced affect host-fungal interactions *in vivo* and *in vitro*, respectively, one would expect that colonization-induced adaptive immunity could reduce susceptibility to systemic candidiasis. Indeed, earlier work by E. Balish’s group already provided evidence for a protective effect of intestinal colonization with *C. albicans* for subsequent systemic infection in mice (Cantorna and Balish, 1991; Wagner et al., 1996) which involved T cells. Huertas et al. (Huertas et al., 2017) confirmed these results and demonstrated a systemic antibody response following colonization, that could provide an explanation for the protective effect. Two recent studies using combined gastrointestinal colonization and tail vein infection not only confirmed these previous observations, but also identified both antibodies and Th17 responses induced by colonization as factors mediating colonization-induced protection against systemic candidiasis.

Shao et al. (Shao et al., 2019) used oral treatment with ampicillin to facilitate intestinal colonization of mice with *C. albicans*. Colonization alone did not result in clinical disease or detectable fungal dissemination into internal organs, but protected animals against systemic challenge. This could be linked to the induction and activation of fungal-specific Th17 CD4^+^ T cells that accumulated in both mucosal and systemic lymphatic tissue. These cells mediate protection by releasing IL-17 which in turn activates neutrophils, which are the most important effector cells for fungal clearance (d'Enfert et al., 2021). Importantly, these immunological changes also decreased susceptibility to the bacterial pathogen *S. aureus*, but increased susceptibility to allergic airway inflammation, highlighting the consequences of the enhanced Th17 response beyond antigen-specific effects. It is also notable that the effect was dependent on persistent fungal colonization. The same authors furthermore provide evidence that the level of *C. albicans* colonization correlates with the frequency of fungal-specific Th17 CD4^+^ T cells and neutrophil reactivity in humans, indicating that these mechanisms are conserved across mice and men.

Using antibody profiling Doron et al. showed that *C. albicans* is the major inducer of antifungal immunoglobulin G (IgG) present in the serum of humans and mice (Doron et al., 2021). Similar to the Th17 responses analyzed by Shao et al., the B cell responses in the study of *Doron et al.* originated in the spleen rather than mucosa-associated lymphoid tissue. The antibodies induced were protective against systemic candidiasis as demonstrated by transfer experiments into B cell-deficient mice.

In addition to adaptive immunity, Tsao et al. (Tso et al., 2018) reported that colonization seems to also induce trained immunity, which is a prolonged phenotypic adaptation of monocytes and macrophages in response to the fungal cell wall components chitin and β-glucan that mediates enhanced protection against systemic challenge (Cheng et al., 2014; Saeed et al., 2014; Rizzetto et al., 2016). Tsao et al. obtained gut-adapted *C. albicans* isolates by repeated long-term passaging through the murine gut. These isolates showed filamentation defects and strongly reduced virulence in the systemic infection model, but facilitated colonization-mediated protection against systemic challenge. As this was also observed in *Rag1* ^−/−^ mice lacking both B and T cells and was accompanied by increased innate cytokine responses, innate-like mechanisms contribute to the protective effect (Tso et al., 2018).

## Discussion

As summarized above, *Candida* colonization is not only one of the risk factors for systemic candidiasis (Eggimann and Pittet, 2014) but also has profound effects on the host immune system that affect susceptibility to systemic candidiasis in mice. This raises further questions, related both to our basic understanding of host-*Candida*-interaction and possible consequences for preclinical animal models.

How are systemic responses induced by colonization? Low-grade dissemination of viable fungal cells is one possibility, and has been observed in *C. albicans*-colonized mice in the absence of clinical symptoms in some studies (Vautier et al., 2015). Instead of viable cells, components of the fungal cell wall could be transported by migrating CX3CR1+ mononuclear phagocytes to distant lymphoid organs (Doron et al., 2021) as described for some bacteria (Zeng et al., 2016). While this can explain the induction of both T and B cell responses, it remains unclear why *C. albicans* is the main inducer of Th17 cells in humans (Bacher et al., 2019) although other yeast such as *Saccharomyces cerevisiae* or *Malassezia restricta* are found even more frequently and with higher prevalence in the human gut (Nash et al., 2017). It is likely that specific features of *C. albicans* promote induction of systemic responses, but it is as of yet unknown what these features are. Intestinal IgA targets *C. albicans* hyphae over yeast (Ost et al., 2021), and hypha formation is an important virulence factor of *C. albicans* epithelial infection (Jacobsen et al., 2012). Comparison of the response to different *C. albicans* strains with different virulence potential could provide insights as to whether occasional, locally restricted epithelial invasion events promote the development of robust adaptive responses to colonization, possibly independent of trained innate immunity that is induced also by a filament-deficient strain (Tso et al., 2018).

Both antibody and Th17 responses require activation of CD4+ T cells, highlighting the importance of these cells for the overall adaptive response to *C. albicans* colonization. But what is the relative contribution of antibodies and IL-17, respectively? Can loss of one mechanism be compensated by the other? These questions cannot be answered yet, but would be clinically interesting regarding risk assessment of patients undergoing specific immunosuppressive therapies. Based on the available data, the beneficial effect of Th17 responses seems to rely on neutrophils (Shao et al., 2019), whereas antibody transfer protected mice treated with cyclophosphamide which induces neutropenia (Doron et al., 2021). This suggests that antibodies protect independent of neutrophils, and that B cell responses complement cellular Th17-driven protection.

How do the new insights on colonization and susceptibility to systemic candidiasis affect the design of infection experiments in mice? So far, immunocompetent SPF mice without any *prior* treatment infected *via* lateral tail vein injection have been used most commonly to study the pathogenesis of systemic candidiasis, to identify virulence factors, and evaluate efficacy of therapeutic approaches. In most cases, this simplistic model is likely to be sufficient for the comparison of fungal deletion mutants to parental strains, or to determine if an antifungal compound is effective *in vivo*. The recent findings are, however, relevant for immunological studies and evaluation of immunomodulatory therapy, because colonized mice and mice that are immunologically naïve to *C. albicans* are likely to respond differently. Shao et al. discuss that contradictory results regarding the role of IL-17A and IL-17 receptor for systemic candidiasis in mice might be the consequence of differences in the occurrence of *C. albicans* as commensal in each facility (Shao et al., 2019). This might also explain discordance of early studies on the role of antibodies for systemic candidiasis in mice (Wagner et al., 1996). Prior exposure to the fungus can even affect results of *ex vivo* models, such as the interaction of *C. albicans* with host cells in a whole-blood model (Machata et al., 2020). Given that human patients with systemic candidiasis are highly likely to have developed adaptive responses before infection, combining colonization with defined tail vein infection could refine this valuable model to better reflect the human situation and to increase the translatability of results from mice to men.

## Author Contributions

Conceptualization and writing of the manuscript were performed by BJ and IDJ. All authors contributed to the article and approved the submitted version.

## Funding

IJ and BJ are supported by the German Research Foundation (DFG) through the TRR 124 FungiNet, “Pathogenic fungi and their human host: Networks of Interaction,” DFG project number 210879364, project C5.

## Conflict of Interest

The authors declare that the research was conducted in the absence of any commercial or financial relationships that could be construed as a potential conflict of interest.

## Publisher’s Note

All claims expressed in this article are solely those of the authors and do not necessarily represent those of their affiliated organizations, or those of the publisher, the editors and the reviewers. Any product that may be evaluated in this article, or claim that may be made by its manufacturer, is not guaranteed or endorsed by the publisher.
